# Reactive granulomatous dermatitis following COVID-19 vaccination

**DOI:** 10.1016/j.jdcr.2024.04.048

**Published:** 2024-07-06

**Authors:** Grecia Figueroa-Ramos, Alejandro Barrera-Godínez, Samantha Paola Bermúdez-Rodríguez, Michelle Gatica-Torres, Judith Guadalupe Domínguez-Cherit

**Affiliations:** a Department of Dermatology, Instituto Nacional de Ciencias Médicas y Nutrición Salvador Zubirán, Mexico City, Mexico; b Department of Dermatology, Boston University, Boston, Massachusetts; c Tecnológico de Monterrey, Mexico City, Mexico

**Keywords:** COVID-19, cutaneous granulomatous reactions, interstitial granulomatous dermatitis, interstitial granulomatous drug reaction, reactive granulomatous dermatitis, vaccine

## Introduction

Granulomatous inflammatory skin conditions exhibit diverse clinical and histologic presentations. Reactive granulomatous dermatitis (RGD) is a comprehensive term that encompasses palisaded neutrophilic and granulomatous dermatitis, interstitial granulomatous dermatitis (IGD), and interstitial granulomatous drug reaction. RGD is typically associated with underlying systemic conditions, including highly reactive immune states (autoimmune diseases and infections), malignancy (hematologic and solid organ), and medications.^1^ These entities show significant overlap in their clinical morphologies, histologic features, underlying causes, and response to treatment. Consequently, they are best regarded as subtypes within this overarching category.^2^

During the COVID-19 pandemic, various postvaccination skin reactions, including granulomatous reactions, have been reported. Here, we discuss a case of an extensive granulomatous eruption following AstraZeneca COVID-19, most consistent with RGD.

## Case report

**Fig 1:**
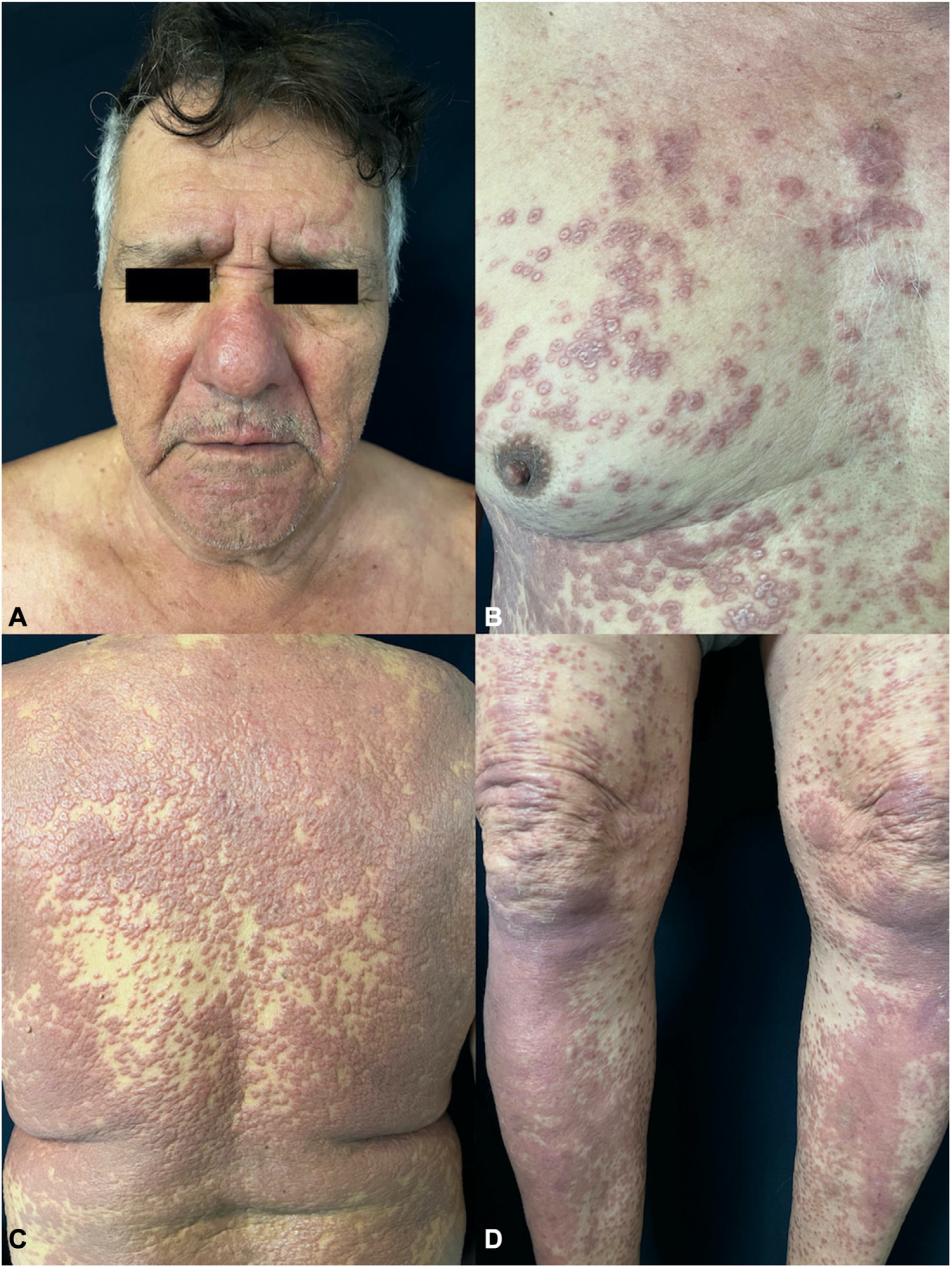
**A-D,** Disseminated confluent annular erythematous plaques with elevated and irregular borders.

A man in his 80s presented with a persistent and widespread pruritic cutaneous eruption that appeared abruptly 1 week after receiving the ChAdOx1-S (recombinant) COVID-19 vaccine (AstraZeneca/Oxford), initially at the vaccination site and then spreading to over 80% of his body. Upon examination, diffuse, confluent annular erythematous plaques with raised irregular borders were observed on his head, neck, trunk, and extremities (Fig 1). Neither hepatosplenomegaly nor palpable lymphadenopathies were found. The rash persisted for 6 months, and the patient experienced associated fatigue and nocturnal fevers. He had not taken any new medications or supplements in the months leading up to the eruption’s onset.

**Fig 2:**
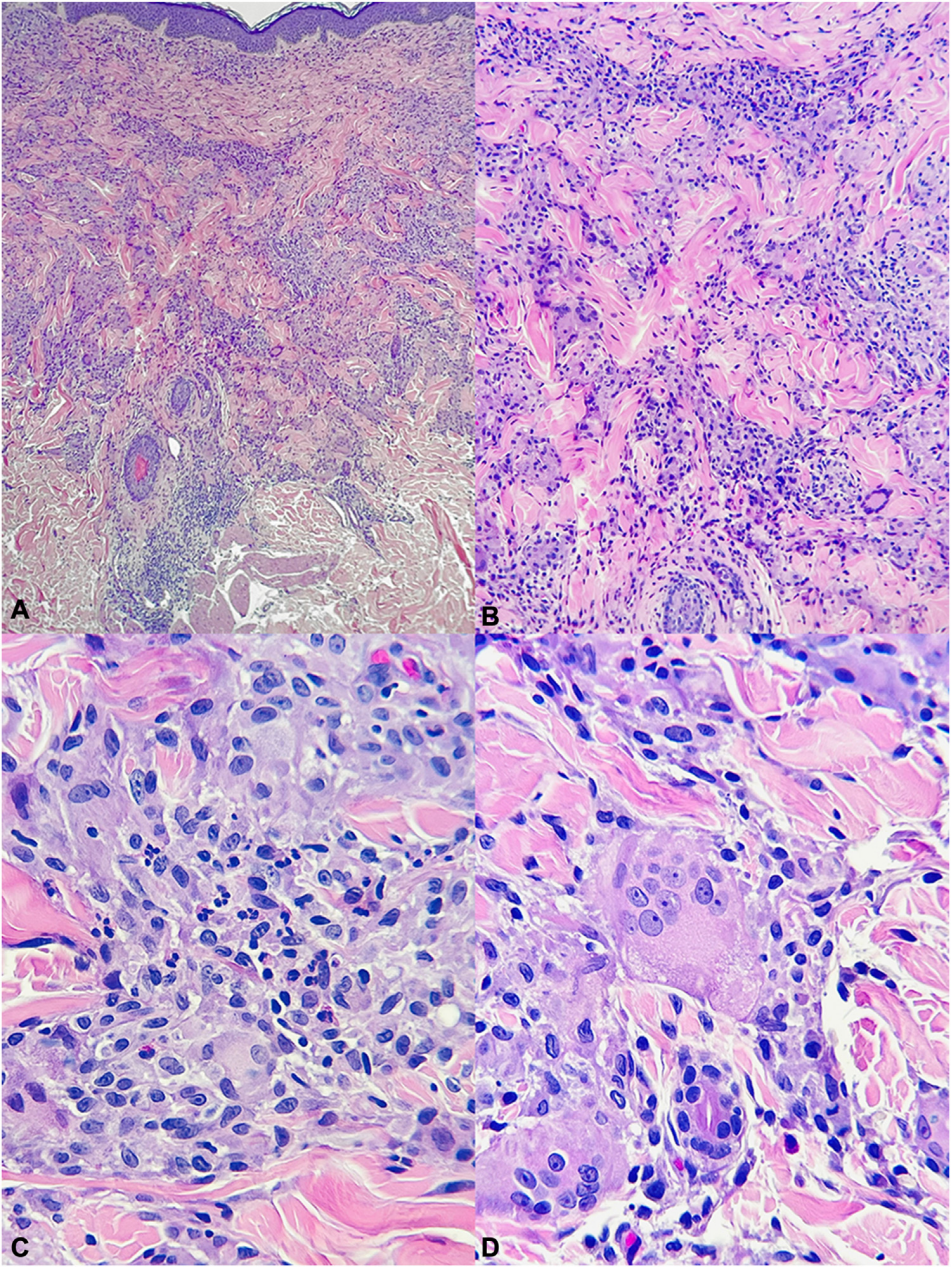
Skin biopsy. **A, B,** An interstitial granulomatous infiltrate on the upper portions of the dermis is observed. **C, D,** The inflammatory cell infiltrate is mainly composed of epithelioid histiocytes and multinucleated giant cells dissecting between collagen bundles and surrounding them (*floating sign*). Admixed neutrophils and eosinophils are also present. (**A-D,** Hematoxylin-eosin stain; original magnifications: **A,** ×40; **B,** ×100; **C** and **D,** ×600.)

Although he initially improved with oral prednisone, relapse occurred during tapering. On account of the extent of skin affected and the associated fever, a skin biopsy was taken and histopathologic sections showed epithelioid histiocytes and multinucleated giant cells dissecting between collagen bundles in an interstitial pattern with scattered neutrophils and eosinophils (Fig 2). Special stains were negative for microorganisms.

**Fig 3:**
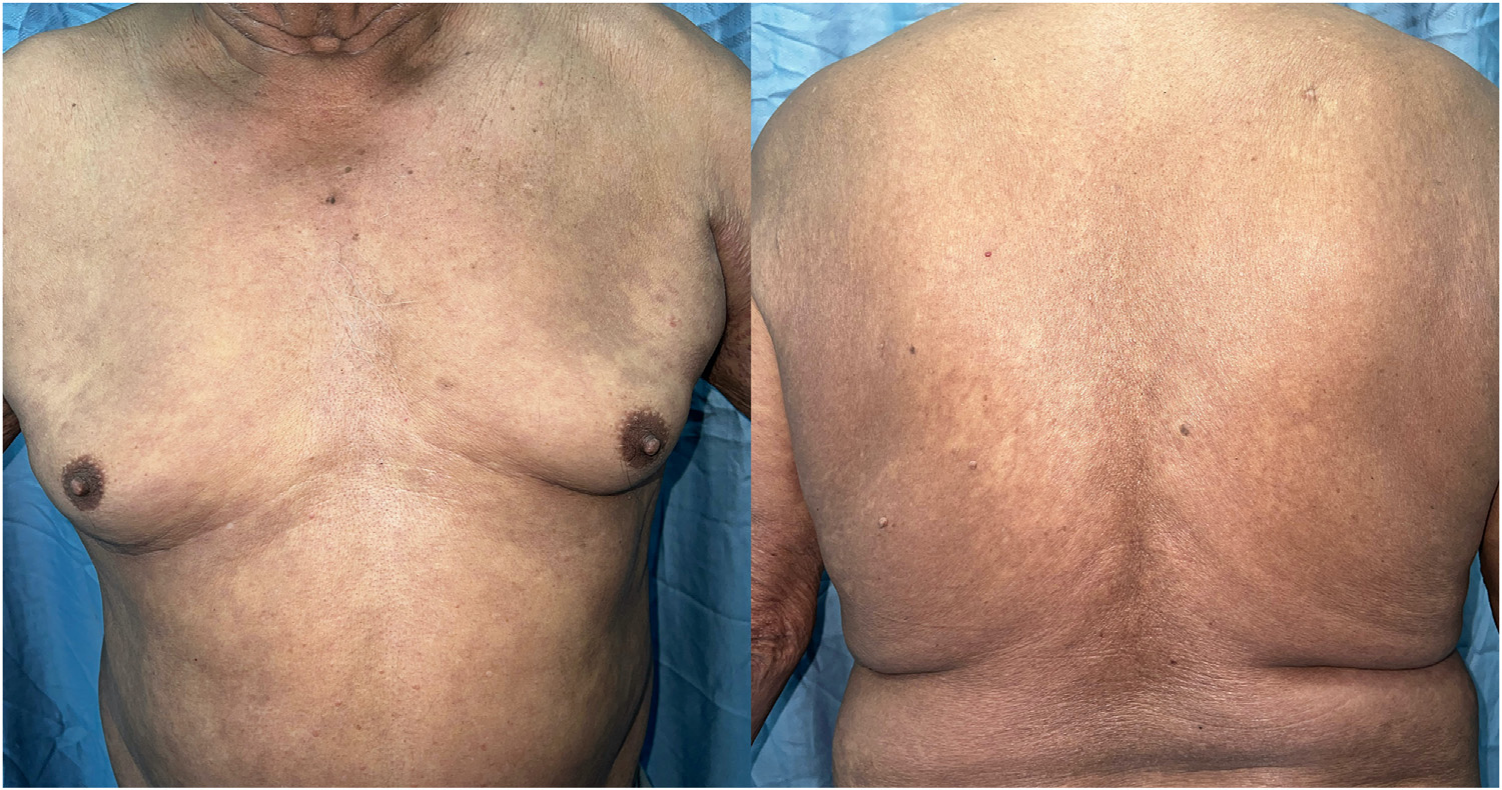
Improvement of the skin lesions, with persistence of postinflammatory hyperpigmentation.

All other possible associated conditions were ruled out (Table I), concluding that the patient presented a RGD associated with COVID-19 vaccination. Treatment with methotrexate (15 mg/wk) and hydroxychloroquine (200 mg/d) was started, leading to significant improvement after 2 months (Fig 3). At 1-year follow-up, he continued to do well. The patient discontinued treatment shortly after, with a recurrence of lesions on his arms and was subsequently lost to follow-up before his second yearly check-up.

**Table I:** Diagnostic approach in our patient

Laboratory testing	
Complete blood count	Monocyte count: 1.14 (normal range: 0.3-0.9) Peak of 24% (normal range: 4.6-13.2)
Procalcitonin	Normal
Urinalysis	Normal.
Viral hepatitis panel	Negative
VDRL	Negative
HIV fast test	Negative
Brucella plate agglutination test	Negative
QuantiFERON-TB	Negative
Anticoccidioides antibodies	Negative
Histoplasmosis ELISA	Negative
Rheumatoid factor	<10 (range: 0-14)
Anti-CCP antibodies	Negative
Antinuclear IgG antibodies (HEp-2-IFI) test	Speckled pattern at 1:160 dilution Mitochondrial pattern at 1:80 dilution
Anti-DNAds antibodies	Negative
Anti-SSa and anti-SSb antibodies	Negative
Anti-smooth muscle antibodies	Negative
HLA-B27	Negative
Prostate-specific antigen	0.47 ng/mL (normal range: 0.03-0.5)
Protein electrophoresis	Unremarkable
C reactive protein	10.82 (normal range: 0-1)
d-Dimer levels	1712 ng/mL (normal range: <500)
Fecal immunochemical test	Negative
Thyroid panel	Normal
Angiotensin-converting enzyme level	44.3 (September 5, 2022), which decreased to 13.1 (March 30, 2023) (normal range: 13.3-63.9)
Imaging testing	
Chest x-ray	No lesions or abnormalities
Head, chest, abdomen, and spine CT scan	No lesions or abnormalities
Full body PET-CT	Generalized irregular skin thickening associated with hypermetabolism, compatible with underlying disease and supradiaphragmatic adenopathies with increased metabolic activity

*CCP*, Cyclic citrullinated peptide; *ELISA*, enzyme-linked immunosorbent assay; *TB*, tuberculosis; *VDRL*, Venereal disease research laboratory.

## Discussion

The term RGD arose due to the need to unify entities with overlapping clinical manifestations, histologic findings, and systemic associations. RGD is a reactive phenomenon influenced by systemic triggers, including connective tissue diseases, inflammatory and reactive arthritides, hematologic malignancies, and medications. Nevertheless, a significant proportion of cases stay classified as idiopathic. The pathogenesis of RGD remains unclear, although a granulomatous response to aberrant deposition of immune complexes has been hypothesized.^1^^,^^2^

Rodríguez-Garijo et al^2^ described a case series of 52 patients with IGD and/or palisaded neutrophilic and granulomatous dermatitis, observing no significant association between IGD and palisaded neutrophilic and granulomatous dermatitis clinical findings, including cutaneous lesions, their localization, and associated symptoms. Bangalore Kumar et al^3^ examined a cohort of 65 patients with RGD with a median age of 62 years (15-81), and although the vast majority of the patients did not exhibit skin-related symptoms, 31.6% cases presented pruritus, and 76.9% had concurrent systemic conditions.

Vaccines have been associated with the induction of localized and generalized granulomatous reactions.^4^ Based on current findings, most skin reactions following SARS-CoV-2 vaccination resemble those occurring during SARS-CoV-2, sharing common immunopathologic mechanisms marked by host immune activation against viral particles rather than direct viral-induced damage.^5^, ^6^, ^7^, ^8^, ^9^, ^10^ The occurrence of granulomatous disease after SARS-CoV-2 raises questions, as there are shared factors in the pathogenesis of both conditions.^6^ It is possible that SARS-CoV-2’s use of angiotensin-converting enzyme II (ACE2) as a receptor, which leads to ACE2 downregulation and angiotensin II accumulation, might trigger ACE upregulation in an attempt to maintain systemic balance. In a pro-Th1 environment, both ACE upregulation and angiotensin II accumulation could stimulate granuloma formation and granulomatous responses.^7^

Polat et al^6^ documented a case involving a woman who experienced sarcoidal granulomas resembling scar sarcoidosis 2 to 3 weeks after presenting COVID-19. They hypothesized that increased interferon gamma expression in CD4^+^ T cells, which has been reported to play a central role in COVID-19-related cytokine storms, may have triggered the activation of adaptive immune responses and initiated the formation of sarcoidal granulomas.^6^

Instances of granuloma annulare have been reported following the administration of SARS-CoV-2 vaccines.^8^ Currently, limited information is available regarding SARS-CoV-2 vaccination-associated RGD. Ariasi et al^9^ reported a case of IGD associated with fever and arthralgias following vaccination with the messenger RNA-1273 SARS-CoV-2 vaccine (Moderna); recurring after booster administration.^9^ Tan et al^10^ reported a case of erythema nodosum and IGD accompanied by fever and nocturnal diaphoresis, 3 days after receiving the second dose of the Pfizer-BioNTech COVID-19 messenger RNA vaccine.^10^

Cutaneous manifestations of RGD are diverse, appearing locally or disseminated to all body segments, and include erythematous papules and nodules, pink papules that coalesce into linear serpiginous cords, annular plaques with central clearing, polycyclic or granuloma annulare-like plaques, subcutaneous linear bands, as well as violaceous patches and plaques.^1^, ^2^, ^3^

Skin biopsies are a valuable diagnostic tool in RGD. Histologic findings involve the presence of histiocytes within the dermis forming palisades, interstitially dissecting between collagen fibers, or forming nodules. Multinucleated giant cells are commonly observed, accompanied on occasion by an inflammatory infiltrate. Additionally, collagen fiber degeneration can be observed in varying proportions.^1^, ^2^, ^3^, ^4^ Differing from classic granuloma annulare, mucin deposition is not observed in RGD.

When clinical and histopathologic findings align with RGD, an extensive evaluation in search of potential systemic associations must be performed, as is common with other reactive dermatoses. Unfortunately, the required diagnostic tools may be lacking, or high costs make a thorough investigation unfeasible.^1^

Treatment aims to control the underlying trigger. Given the rarity of this condition, there is no established treatment consensus. However, responses have been observed with topical and systemic corticosteroids, hydroxychloroquine, colchicine, methotrexate, cyclosporine, cyclophosphamide, intralesional corticosteroids, and dapsone. Biologics agents such as etanercept have even been used for refractory cases. Spontaneous remission has also been documented in some cases.^1^, ^2^, ^3^, ^4^^,^^9^^,^^10^

## Conflicts of interest

None disclosed.
